# Assessment of Habitat Suitability for Amphioxus in the Changli Marine Reserve and Adjacent Coastal Waters, Hebei Province

**DOI:** 10.3390/ani15213203

**Published:** 2025-11-03

**Authors:** Yongfeng Zhang, Qiuzhen Wang, Quanying Wang, Qianqian Zhao, Weijie Shi, Yong Zhang, Yuan Yao, Jianle Zhang

**Affiliations:** 1 Qinhuangdao Marine Center, The Ministry of Natural Resources, Qinhuangdao 066000, China; qhdhyzyf@126.com (Y.Z.);; 2 Ocean College, Hebei Agricultural University, Qinhuangdao 066000, China

**Keywords:** amphioxus, habitat suitability, HSI, marine reserve, AHP, spatiotemporal analysis, environmental stressors

## Abstract

**Simple Summary:**

Amphioxus is a rare marine species that provides important clues about the origin of vertebrates and serves as a sensitive indicator of coastal ecosystem health. However, current research on amphioxus has been limited, often focusing only on abundance data from single years or specific sites, without linking population changes to environmental conditions. Studies have also rarely addressed long-term patterns or considered the combined effects of natural and human disturbances. These gaps make it difficult to understand the survival challenges of amphioxus and to design effective conservation strategies. In this study, we analyzed over a decade of observations from the coastal waters of Changli, Northern China. By linking population trends with environmental factors, our research offers new insights into habitat conditions and risks facing amphioxus. The findings highlight the value of long-term monitoring and provide scientific support for protecting marine biodiversity and ensuring the sustainable management of sensitive coastal habitats.

**Abstract:**

Amphioxus, a key model organism in vertebrate evolution, is essential for understanding ecological balance and species diversity. This study examined the spatiotemporal distribution of *Branchiostoma japonicum* and assessed its habitat suitability in the Changli Golden Coast Nature Reserve and adjacent coastal waters from 2008 to 2023 (excluding 2020). The maximum abundance showed marked fluctuations, with a sharp decline between 2008 and 2015 followed by recovery after 2016, reaching a peak of 345 ind./m^2^ in 2022. The average abundance also increased, peaking at 34 ind./m^2^ in 2022. Spatially, the higher abundances occurred in central stations, while peripheral sites were much lower, sometimes absent. Spearman’s correlation and Principal Component Analysis (PCA) identified sediment grain size (1~0.25 mm), water depth and sediment sulfide as key environmental factors. A habitat suitability index (HSI), constructed using the Analytic Hierarchy Process (AHP), showed higher values in central stations, indicating more favorable conditions. These findings highlight the importance of long-term monitoring, clarify the ecological requirements of amphioxus, and provide guidance for habitat conservation and management in regions affected by environmental change and human activities.

## 1. Introduction

The Hebei Changli Golden Coast National Nature Reserve represents a typical marine-terrestrial interface in northern China, situated in the western part of Bohai Bay. It serves as a crucial area for studying coastal ecosystems and marine biological resources. Within the reserve, there exist both high-quality sandy beaches and abundant marine biodiversity, including amphioxus *Branchiostoma japonicum* (classified as a second-grade national protected wild animal in China), which is recognized as a vital model organism for studying vertebrate evolution [1,2,3]. The reproductive characteristics and resource dynamics of *B. japonicum* exhibit pronounced seasonality and instability. Reproduction shows clear seasonal patterns, typically commencing when water temperatures exceed 23 °C, and spawning frequency can be modulated by temperature regulation [4,5]. However, although temperature control has been demonstrated to influence spawning frequency, the precision and reliability of this approach require further validation under varying environmental conditions [5]. Moreover, a mass reproductive event that occurred in 2002 on the Ohzu sandbank in the western Seto Inland Sea of Japan markedly altered the long-term population structure [6]. While the monitoring of this event spanned four years and provided valuable insights into population dynamics, the data remain insufficient for understanding long-term population changes and environmental adaptability [6]. Future studies should further investigate the environmental triggers of reproduction in *B. japonicum* and explore how precise environmental regulation can be leveraged to achieve sustainable resource management, which is essential for conserving this important species and maintaining ecosystem stability [7]. To effectively manage *B. japonicum* resources, long-term monitoring programs should be established to track population dynamics, while simultaneously protecting and restoring critical habitats to ensure the stability of their survival and reproductive environments.

Amphioxus typically inhabit shallow bottoms composed of sandy or muddy sediments, with water depths generally below 20 m with fine sand grains rich in organic matter [8,9,10,11]. The intertidal and shallow zones of the Changli Reserve possess the basic conditions for amphioxus survival, including clean sandy seabeds and relatively stable environmental conditions such as temperature and dissolved oxygen [12,13]. Additionally, while amphioxus predominantly inhabit exposed sandy areas, seagrass beds also serve as important habitats for them [14]. Amphioxus primarily feeds through filtration, relying on phytoplankton, zooplankton and organic debris in seawater, with eutrophication or pollution potentially having severe impacts on their habitats [15,16,17].

In recent years, the amphioxus habitats within the Changli Reserve have faced multiple pressures, leading to significant changes in their distribution. Coastal development, urbanization and industrialization have caused damage to the intertidal and shallow sea areas, directly reducing the suitable habitat for amphioxus. The construction of water conservancy projects upstream of the Luan River has decreased the supply of suspended sediments entering the sea, resulting in the degradation of the River Delta and further threatening the amphioxus habitat [1]. Meanwhile, large-scale scallop farming has increased competition for food resources, reducing feeding opportunities for amphioxus [1,18]. Changes in sediment composition could directly affect the burrowing behavior and survival rate of amphioxus by altering the stability and pore structure of the substrate [19]. This mechanism of sediment-biota interaction is crucial in habitat assessment. Additionally, marine pollution, eutrophication and sediment contamination from industrial discharges pose threats to amphioxus populations [16,19]. Ocean temperature rise due to climate change may not only shift the physiological tolerance limits of amphioxus populations [20]. But sea-level changes could also indirectly alter habitat substrate structure by reshaping hydrodynamic conditions. Furthermore, the potential impacts of emerging pollutants may continuously exacerbate environmental pressures on these habitats [21]. It is noteworthy that current research on amphioxus lacks a comprehensive habitat suitability assessment framework. To address escalating threats from coastal urbanization and marine pollution, this study establishes a habitat suitability framework for amphioxus to guide evidence-based conservation of this ecologically pivotal species.

This study aims to conduct an in-depth analysis of the amphioxus habitats within the Hebei Changli Golden Coast National Nature Reserve and its adjacent sea areas, to assess the habitat suitability of this species. By collecting and analyzing environmental monitoring data from the period 2008 to 2023 (excluding 2020), with a focus on key environmental factors such as water quality, sediments and particle size, as well as combining these data with changes in amphioxus abundance, this study focuses on identifying the main indicator factors affecting habitat quality. Additionally, this research intends to develop a Habitat Suitability Index (HSI) and evaluate the suitability of different sampling stations, in order to reveal the survival status and distribution patterns of amphioxus under varying environmental conditions. The results of this study will provide a scientific basis for effective conservation and habitat restoration efforts for amphioxus within the Changli Reserve.

## 2. Materials and Methods

### 2.1. Study Area

**Figure 1:**
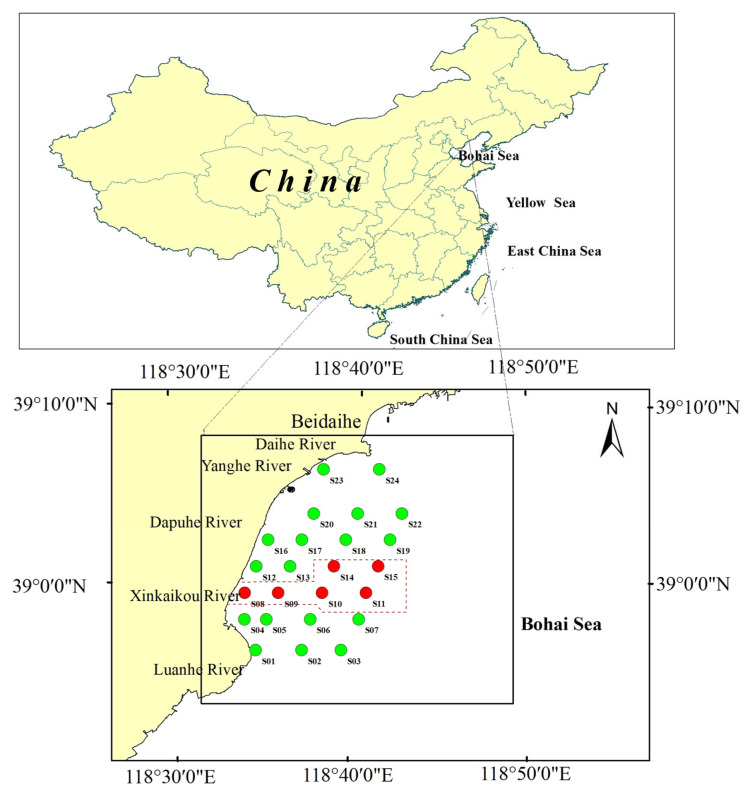
Sampling stations. Stations marked with red circles are located within the Changli Reserve, while those marked with green circles are located outside the reserve.

The study area covers approximately 900 km^2^, located within the Changli National Nature Reserve in Hebei Province, Northern China, and its surrounding coastal waters, specifically spanning from the Yanghe Estuary to the Luanhe Estuary (Figure 1). A total of 24 sampling stations were set up in the study area. The distances between the stations range from 3.5 to 10 km. The stations within the protected area include primarily S8, S9, S10, S11, S14 and S15, while the remaining stations are located outside the protected area. The Changli Reserve primarily protects the natural sandy coastal landscape, which includes dunes, sandbars, lagoons, forest belts and marine organisms. Aquaculture activities are strictly prohibited within the reserve, but scallop farming is distributed outside the protected area, overlapping all the stations outside the reserve.

### 2.2. Data Collection

Monitoring data were collected during summer (August) from 2008 to 2023 in the Changli Golden Coast Nature Reserve and its adjacent coastal areas, following the Marine Monitoring Specification [22]. The year 2020 was excluded from the dataset due to unsampled conditions resulting from COVID-19 pandemic restrictions. These data were organized and summarized to include various environmental parameters from annual surveys of amphioxus abundance, water quality and sediments (Table S1). Water quality parameters encompassed temperature, depth, salinity (S), suspended solids (SS), pH, dissolved oxygen (DO), chemical oxygen demand (COD), dissolved inorganic phosphorus (DIP), nitrite (NO_2_^−^), nitrate (NO_3_^−^), ammonia nitrogen (NH_4_^+^), dissolved inorganic nitrogen (DIN), chlorophyll-a (Chl-a), transparency, oil, silicate (Si), copper (Cu), lead (Pb), cadmium (Cd), zinc (Zn), chromium (Cr), mercury (Hg) and arsenic (As). Sediment parameters included total nitrogen (S-TN), total phosphorus (S-TP), silicon (S-Si), total organic carbon (S-TOC), sulfide (S-sulphide), petroleum (S-oil), mercury (S-Hg), arsenic (S-As), copper (S-Cu), lead (S-Pb), cadmium (S-Cd), zinc (S-Zn) and chromium (S-Cr). Sediment grain size parameters comprised grain size content (Grain 1~0.5 mm, Grain 0.5~0.25 mm, Grain 0.125~0.063 mm and Grain < 0.001 mm), grain group content (sand, silty sand and clay), and grain group coefficients (Md φ, QD φ and SK φ). Additionally, the sum of sediment grain sizes in the 1~0.5 mm (Grain 1~0.5 mm) and 0.5~0.25 mm (Grain 0.5~0.25 mm) ranges was considered as a new environmental parameter, termed sediment grain size 1~0.25 mm (Grain 1~0.25 mm). The sum of silt and clay contents was also designated as a new parameter, namely silty and clay. In total, these 50 environmental parameters were sourced from annual monitoring data provided by the Qinhuangdao Marine Center of the Ministry of Natural Resources.

Sediment samples were collected for the abundance analysis of amphioxus. When collecting sediment samples, a 0.05 m^2^ sediment sampler was used, and three samples were taken at each station. Under special circumstances, the number of samples taken shall not be less than two times. The abundance of amphioxus was calculated based on the number obtained. In addition, *B. belcheri tsingtauense* is currently treated as a synonym of *B. japonicum*, following recent taxonomic revisions, and the identification was based on morphological traits [1]. Amphioxus are characterized by their translucent, lanceolate body, persistent notochords, and V-shaped myomeres, features absent in worms (Figure S1). In addition, amphioxus abundance was calculated based on replicate quadrat sampling at each station.

### 2.3. Screening of Habitat Indicators

In this study, 50 water quality and sediment environmental parameters from the summer months of the Changli Golden Coast Nature Reserve and its adjacent coastal areas between 2008 and 2023 (excluding 2020) were selected as environmental factors influencing the habitat of amphioxus. Initially, Spearman’s correlation analysis was employed to screen out factors with extremely significant impacts on amphioxus abundance (*p* < 0.01), while factors with weak correlations (*p* > 0.01) were removed. Subsequently, for the environmental factors identified in the previous step, the Kaiser-Meyer-Olkin (KMO) value was calculated, and Bartlett’s Test of Sphericity was conducted to exclude factors that did not meet the criteria (KMO < 0.6). Thereby, the remaining factors suitable for Principal Component Analysis (PCA) were selected. Finally, PCA was conducted using these remaining factors to identify key environmental factors with substantial and independent contribution rates, which were then designated as habitat indicator factors. All the aforementioned analyses were performed using R software version 4.3.1 and the FactoMineR package version 2.11.

### 2.4. Modeling of Habitat Fitness Curve

Currently, there are three primary methods for establishing habitat suitability curves, i.e., referencing expert opinions or related literature, utilizing habitat use models and employing habitat preference models [23,24]. Among them, the expert-literature model relies on experience rather than data, leading to significant subjective influence. The preference model involves overly complex operations. Whereas the use model is directly derived from measured data, offering high usability and operability. Therefore, the habitat use model was selected in this study.

### 2.5. Calculation of Habitat Suitability Index (HSI)

Considering the varying impacts of different habitat factors on the amphioxus population, the weighted average method was proposed to calculate HSI.

HSI = k1∗f1 + k2∗f2 + … + ki∗fi

In the formula, ki represents the weight of the ith habitat factor, and fi represents the suitability of the ith habitat factor, indicating the degree of suitability for that factor, with a threshold value ranging from 0 to 1. The Analytic Hierarchy Process (AHP) was utilized to determine the weights of the factors [25]. The AHP method constructs a hierarchical structure based on the dominance relationships among the factors and determines the weights through a unified combination of qualitative and quantitative analyses. The calculation of HSI is completed using R software version 4.3.1.

## 3. Results

### 3.1. Analysis of Amphioxus Abundance

**Figure 2:**
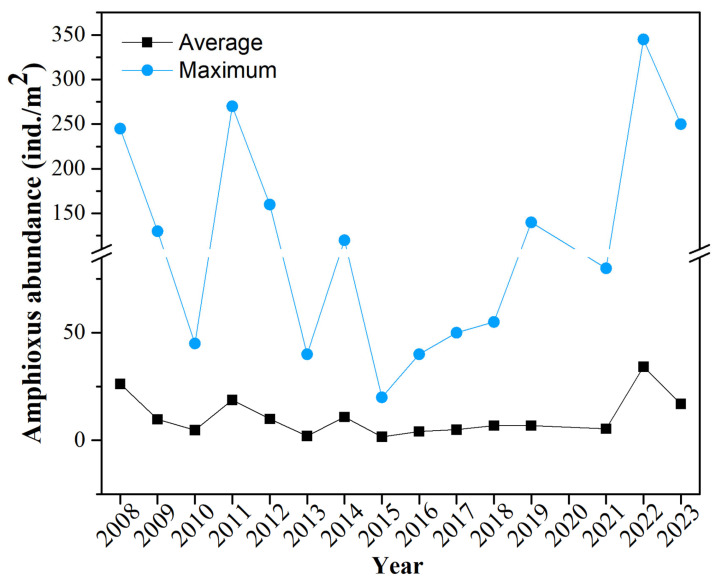
Interannual variation in the maximum and average abundance of amphioxus from 2008 to 2023 (Excluding 2020). The abundance of amphioxus refers to the number of amphioxus individuals obtained per unit sampling area. The maximum abundance of amphioxus refers to the maximum value of amphioxus abundance among all sampling stations in a specific year. The average abundance of amphioxus refers to the average value of amphioxus abundance across all sampling stations in a specific year.

The population abundance of amphioxus experienced obvious fluctuations between 2008 and 2023 (Figure 2). The maximum abundance gradually recovered after large fluctuations from 2008 to 2015 and then increased sharply between 2021 and 2022. While the average abundance showed a relatively small but overall increasing trend. During the period from 2008 to 2023, the average abundance was 11 ind./m^2^, with a maximum abundance of 345 ind./m^2^ and a minimum abundance of 0. Throughout the investigation period, the maximum abundance of amphioxus exhibited considerable fluctuations, particularly after significant variations from 2008 to 2015, followed by a gradual recovery starting in 2016 and ultimately a notable increase between 2021 and 2022. In contrast, the average abundance varied relatively little, indicating substantial differences in the distribution and population abundance of amphioxus across different stations. From 2008 to 2015, the maximum abundance peaked significantly in 2011 and 2014, with values of 270 ind./m^2^ and 120 ind./m^2^, respectively. However, an overall trend of fluctuating decline was observed, especially with a larger decrease in 2015. Between 2016 and 2019, the maximum abundance gradually increased initially in 2016 and reached a small peak in 2019 (140 ind./m^2^), with an insignificant increase in average abundance at this time. From 2021 to 2023, the maximum abundance rose sharply from 2021 to 2022, reaching its highest value in 2022 (345 ind./m^2^). Although it declined slightly in 2023, the abundance remained at a high level. Between 2021 and 2022, a coastal ecological-restoration initiative, “mariculture withdrawal and habitat restoration”, was implemented next to the Changli reserve, removing large-scale aquaculture operations. The sharp surge in amphioxus abundance recorded in 2022 most likely reflects the benefits of this reduction, which markedly diminished aquaculture effluent and benthic disturbance improved the sandy bottom habitat, fostering the population rebound. The average abundance also reached a higher peak in 2022, at 34 ind./m^2^, which is 5.3 times higher than before the restoration. In summary, based on the interannual trend of amphioxus abundance, the population experienced considerable fluctuations within the study area and showed a clear recovery trend in recent years, with abundance peaking in 2022, indicating improved growth conditions for the population in this region.

**Figure 3:**
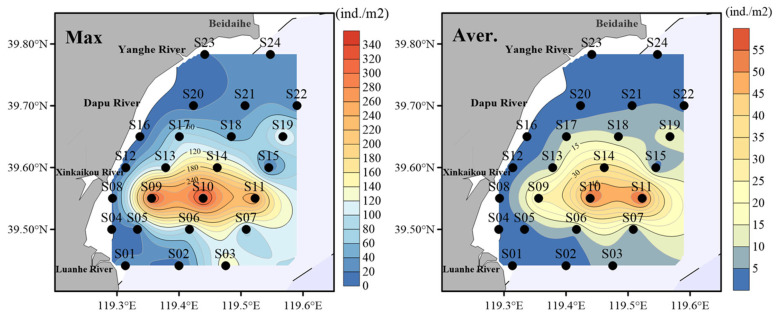
Spatial distribution of the maximum (Max) and average (Aver.) abundance of amphioxus from 2008 to 2023 (excluding 2020). The abundance of amphioxus refers to the number of amphioxus individuals obtained per unit sampling area. The maximum abundance of amphioxus refers to the maximum abundance of amphioxus at a specific station from 2008 to 2023 (excluding 2020). The average abundance of amphioxus refers to the average abundance of amphioxus at a specific station from 2008 to 2023 (excluding 2020).

From a spatial distribution perspective, the abundance of amphioxus is relatively concentrated, exhibiting an overall trend of gradually decreasing from the center to the periphery (Figure 3). On the one hand, the maximum abundance of amphioxus is primarily concentrated at stations S09, S10 and S11 within the Changli Reserve, with maximum abundances of 335, 345 and 270 ind./m^2^, respectively, indicating significant high-density aggregation areas. Other stations such as S03, S13, S14 and S19 also have relatively high abundances, all exceeding 100 ind./m^2^. However, in the southern (near S04, S05) and northern (near S20, S21, S22) regions around the Changli Reserve, abundances are lower, falling below 100 ind./m^2^, presenting a low-density distribution. Notably, the abundances of amphioxus at stations S04 and S16 are zero.

On the other hand, the distribution of the average abundance of amphioxus is similar to that of the maximum abundance but at significantly lower overall levels (Figure 3). The highest average abundances are observed near stations S10 and S11 within the Changli Reserve, with values of 56 ind./m^2^ and 59 ind./m^2^, respectively. Stations near S09 and S14 also exhibit relatively high average abundances, at 26 and 33 ind./m^2^, respectively, suggesting that this area is the primary distribution center for amphioxus. In the marginal waters of the Changli Reserve, such as at stations S06, S07, S13, S19, and S21, the average abundance of amphioxus is lower, below 10 ind./m^2^, indicating that these areas may not be primary habitats for them. Furthermore, in the more outer marginal areas, such as at stations S01, S05, S08, S15, S20, S23 and S24, the abundance of amphioxus is below 5 ind./m^2^, suggesting that environmental conditions in these regions may limit the distribution of amphioxus, resulting in sparse populations.

From the general distribution trend of amphioxus, no matter the maximum abundance or the average abundance, the distribution of amphioxus was concentrated in the protected area (Figure 3). This suggests that these regions likely serve as primary habitats for amphioxus or possess more favorable ecological conditions. Lower abundances in marginal areas indicate that the distribution of amphioxus exhibits strong spatial heterogeneity within the study area, potentially associated with marine environmental factors such as water depth, sediment type or current conditions. For example, benthic biodiversity decline was shorter and milder in the open, well-flushed Dadeng Sea than in the semi-enclosed, sediment-trapping Tongan Bay, showing that hydrodynamics and openness govern both the degree and duration of impact [26]. These differences in distribution patterns also reflect the aggregative behavior and habitat selection characteristics of amphioxus populations.

### 3.2. Habitat Indicator Factor Analysis

Initially, a Spearman Correlation Analysis was conducted between various environmental factors and amphioxus abundance (Table 1). The results indicated that amphioxus abundance exhibited a highly significant correlation (*p* < 0.01) with 18 environmental factors, encompassing sediment organic carbon, sulfides, petroleum hydrocarbons, different particle size fractions (1~0.5 mm, 0.5~0.25 mm, 1~0.25 mm, 0.125~0.063 mm and <0.001 mm), sand content, silt content, clay content, as well as water quality parameters such as water depth, chemical oxygen demand (COD), nitrite, nitrate, inorganic nitrogen, chlorophyll-a and transparency (Table 1). Specifically, amphioxus abundance showed a highly significant positive correlation (*p* < 0.01) with particle size fractions of 1~0.5 mm, 0.5~0.25 mm and 1~0.25 mm, as well as sand content, water depth and transparency. Conversely, it exhibited a highly significant negative correlation (*p* < 0.01) with sediment organic carbon, sulfides, petroleum hydrocarbons, particle size fractions of 0.125~0.063 mm, <0.001 mm, silt content, clay content, as well as water quality parameters including COD, nitrite, nitrate, inorganic nitrogen and chlorophyll-a. Therefore, these 18 environmental factors were selected for further analysis, while other unrelated factors were excluded.

Subsequently, PCA screened 8 factors including Depth, Grain 0.125~0.063 mm, Grain < 0.001 mm, Grain 1~0.25 mm, Silty sand, S-sulphide, NO_3_ and DIN. Then, based on the previous research experience and literature review, three key environmental factors were selected from the 18 environmental factors. Numerous studies have indicated that substrate type is a crucial factor influencing the distribution of amphioxus, which primarily inhabit substrates ranging from sand to fine sand [1,11,17,19]. Consequently, the particle size fraction of 1~0.25 mm (Grain 1~0.25 mm) was chosen as an important factor affecting amphioxus distribution. Based on practical sampling experience, it was observed that amphioxus typically inhabit shallow coastal areas, suggesting that water depth is also one of the significant factors influencing their distribution [17]. Furthermore, large-scale scallop aquaculture is present in the study area. Sulfur-containing organic matter in scallop feces could produce sulfides through anaerobic decomposition. In this study, sulfide content was significantly negatively correlated with the amphioxus abundance. Similarly, it is reported that sulfide content was negatively correlated with benthic biomass [1]. Sulfide stress can lead to symptoms such as growth retardation, anorexia and weakened constitution in amphioxus, subsequently impacting their reproductive capacity and population size [27,28]. Considering the completeness of historical data, sediment sulfide (S-sulphide) was selected as the third key factor due to its highly significant impact (*p* < 0.01) on amphioxus abundance.

### 3.3. Construction of Habitat Suitability Curves

**Figure 4:**
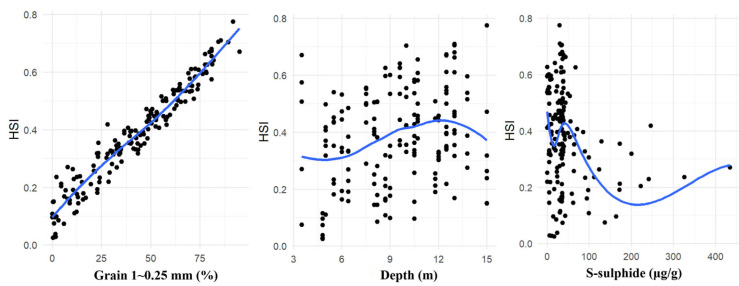
Habitat suitability curves of amphioxus abundance for sediment grain size 1~0.25 mm (Grain 1~0.25 mm), water depth and sediment sulphide content (S-sulphide).

To construct habitat suitability curves using habitat use models, the frequency distributions were applied to correlate with amphioxus abundance based on the collected data. Single-factor suitability curves were established for three habitat indicator factors, i.e., sand grain size fraction of 1~0.25 mm (namely Grain 1~0.25 mm), water depth and sediment sulfide content (Figure 4). The variation range of the Grain 1~0.25 mm across stations was 0–94.94%. Within this range, the HSI values for amphioxus increased linearly with an increase in it. The water depth varied between 3.5 m and 15.0 m across stations. Within this range, the HSI values for amphioxus generally increased first and then decreased with an increase in it, peaking at around 12 m. Additionally, the sediment sulfide content ranged from 0 to 434 μg/g across stations. Within the lower concentration range, the HSI values exhibited a fluctuating decrease with an increase in sediment sulfide content, reaching the lowest point at approximately 210 μg/g of sulfide. This could be attributed to the relatively small negative impact on the amphioxus habitat at lower S-sulfide concentrations. And amphioxus may adapt to such environments by adjusting their physiological functions or behavioral habits. As sulfide content gradually increases, it may exert greater impacts on their life activities such as respiration, feeding and reproduction, leading to a gradual decline in the HSI values. However, when sediment sulfide content reaches higher levels, the HSI values increase slowly again. This phenomenon may be attributed to the adaptability of amphioxus to the environment and the combined effects of multiple environmental factors. However, no significant negative correlation (*p* > 0.05) between the amphioxus density and the sulfide content in sediment was observed in the same research area from 1999 to 2011 [1]. Notably, in this study, the sulfide content in sediments met the second-grade marine sediment quality standard, whereas the referenced research reported sulfide levels compliant with the first-grade standard (≤300 μg/g). These underscore the synergistic effects of multi-stressor interactions (e.g., hypoxia, warming) and their long-term impacts on habitat suitability.

### 3.4. Habitat Suitability Index (HSI) and Its Relationship with Amphioxus Abundance

The weights of the indicator factors were determined using the Analytic Hierarchy Process (AHP) method (Table 2). A discrimination matrix for the indicator factors was constructed based on relevant literature, expert opinions and sampling data. Considering that the habitat of amphioxus typically comprises medium to fine sand bottom substrates, the highest weight was assigned to the content of Grain 1~0.25 mm. Amphioxus generally inhabits shallow waters. Therefore, water depth also has a significant impact on amphioxus, receiving a weight second only to that of Grain 1~0.25 mm. S-sulfide has a potential impact on amphioxus, primarily affecting its respiratory and circulatory systems, nervous system as well as its survival and reproduction. Hence, S-sulfide was selected as the indicator factor with the smallest weight. Based on this, an indicator factor judgment matrix was constructed, with a Consistency Ratio (CR) value of 0.00 [25]. This value is less than 0.1, indicating that the judgment matrix passes the consistency test. This means that the weight distribution is logical and can effectively reflect the impact of various environmental factors on amphioxus habitats. The weights for Grain 1~0.25 mm, water depth and S-sulfide were determined to be 0.65, 0.22 and 0.13, respectively, which were calculated using the geometric mean method.

Finally, the HSI for each station was calculated using the weighted average method with the average value of a single year (Table 3). Overall, the stations within the Changli Reserve (S10, S11 and S14) exhibited high HSI values, indicating that the habitat conditions at these stations are relatively suitable for the survival of amphioxus. Stations located in the vicinity of the reserve (such as S07, S17, S18 and S19) followed suit. In contrast, the coastal stations far from the reserve (S02, S04 and S20) had lower HSI values. Among them, the highest HSI values were observed at stations S10 and S11, with a maximum of 0.52, suggesting that the environmental factors at these two stations are extremely favorable for amphioxus. Stations S18 and S19 followed closely, both having an HSI value of 0.51. The lowest HSI value was recorded at station S04, which was 0.06.

**Figure 5:**
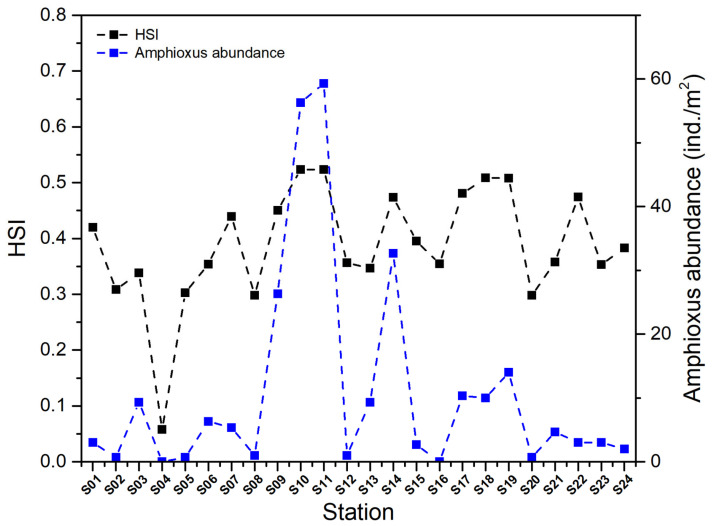
Relationship between the habitat suitability index (HSI) and amphioxus abundance with the fitting coefficient (R2) at 0.34 (*p* < 0.01).

Overall, the HSI of amphioxus across different stations generally aligns with their abundance distribution, although there is some mismatch between HSI values and amphioxus abundance at certain stations (Figure 5). This discrepancy can be attributed to the prolonged data collection period spanning from 2008 to 2023, during which substantial environmental variations took place within the surveyed region, resulting in multiple relocations of amphioxus abundance distribution and aggregation zones. The HSI values at different stations exhibited variations over time, which in turn affected the correspondence between HSI and abundance distribution throughout the monitoring period. For instance, amphioxus was detected at station S01 in both 2008 and 2017 but was absent in other years. Many studies assessing the habitat suitability and influencing factors of amphioxus often adopt a shorter time scale [1,17,29]. However, considering the impact of human activities on amphioxus habitats and the potential changes in their aggregation sites following environmental restoration, a research perspective that encompasses a longer time scale is more conducive to comprehensively identifying potential habitats suitable for their survival and effectively evaluating their ecological recovery. In addition, the discrepancy between HSI and amphioxus abundance primarily stems from three factors, including model limitations (e.g., static assumptions, weight bias), data issues (spatiotemporal scale mismatch) and ecological complexity (larval recruitment and density dependence). Future improvements should incorporate dynamic calibration and multi-scale data integration to enhance prediction accuracy.

## 4. Discussion

Amphioxus, as a pivotal model organism in vertebrate evolution, plays a crucial role in maintaining species diversity and ecological balance [30,31]. In recent years, the Changli Reserve and its surrounding areas have faced multiple ecological pressures, such as coastal development, scallop aquaculture, and damming upstream of the Luan River, all of which pose threats to the amphioxus habitat. Studies have shown that even marginal variations in benthic substrate characteristics and physicochemical water parameters can profoundly influence the spatial distribution patterns and population density of amphioxus [17]. Therefore, science-based, targeted coastal zone management strategies are imperative for the conservation of this ecologically significant chordate species. This study systematically establishes, for the first time, an assessment system for amphioxus habitats in the Changli coastal area and quantifies the impact of different environmental factors on amphioxus populations, aiding in the identification of key habitat indicator factors and potentially threatened areas.

Although our Habitat Suitability Index (HSI) model effectively captured habitat suitability patterns during the study period, we observed a certain mismatch between HSI predictions and actual amphioxus abundance. This mismatch may be related to certain ecological complexities in the amphioxus life cycle that are not fully considered by the current HSI model. For example, the larval dispersal capability significantly affects the distribution and abundance of amphioxus. At some sites, despite high HSI values, the actual abundance may be lower than expected due to insufficient larval dispersal. Recruitment variability is also an important factor influencing amphioxus population dynamics; changes in environmental conditions can lead to significant increases or decreases in recruitment numbers. Additionally, density-dependent mechanisms may affect the distribution and abundance of amphioxus, with high-density populations potentially experiencing intensified resource competition, thereby limiting further population growth. To better understand and explain the mismatch between HSI predictions and actual abundance, future research could consider incorporating ecological complexities such as larval dispersal, recruitment variability, and density dependence into the HSI model. Moreover, adopting a multi-method approach or cross-validation (for example, combining the Analytic Hierarchy Process (AHP) with the entropy weight method, and comparing the results between the MaxEnt and GLMs) can overcome the limitations of a single method and enhance the scientific rigor and reliability of the evaluation results.

Aquaculture activities, particularly scallop farming, significantly affect the habitat of amphioxus by increasing organic matter in sediments and altering local hydrodynamic conditions. These changes may create hypoxic conditions in the sediments, thereby influencing the survival and reproduction of amphioxus. In addition, dam construction in rivers reduces sediment supply and alters water chemistry, exerting negative impacts on amphioxus habitats. Such changes may affect the physiological adaptability of amphioxus, consequently influencing their distribution and abundance. To more comprehensively assess the impacts of these human activities, future research should integrate long-term monitoring data and adopt multidisciplinary approaches to further quantify their specific effects on sediment and water conditions. These efforts will render our study more comprehensive and in-depth, providing stronger scientific support for the conservation and management of amphioxus.

In the Changli Reserve, the habitat selection of amphioxus is primarily influenced by sediment grain size, water depth and sulfide concentration. These factors exhibit both similarities and differences among amphioxus populations in different regions globally. In subtropical Hong Kong waters, the amphioxus species (*B. belcheri*, *B. japonicum* and *B. malayanum*) prefer shallow water areas and coarse sand substrates (especially sand with a grain size of 1~0.25 mm), which facilitate their filter-feeding and respiration [17]. However, an increase in sulfide content may be toxic to amphioxus, affecting their survival and reproduction [17]. In the western Seto Inland Sea, a significant recruitment event of *B. japonicum* larvae in 2002 led to a substantial increase in the population, highlighting the importance of larval supply for population dynamics [6]. Sediment grain size and water depth also affect the habitat and survival of amphioxus, with coarse sand substrates aiding their filter-feeding and respiration [6]. In the southern Gulf of California, *B. californiense* has been found not only in exposed sandy areas but also in seagrass beds, which provide additional habitats for reproduction and feeding, thus increasing habitat diversity [14]. In the Tuscan Archipelago (Western Mediterranean Sea), *B. lanceolatum* is mainly affected by human activities such as sand extraction, which leads to habitat destruction and a significant decrease in amphioxus numbers [19]. Additionally, sediment grain size and organic pollution levels impact the survival and reproduction of amphioxus, with coarse sand substrates facilitating filter-feeding and respiration, while low organic pollution levels indicate a less polluted habitat [19]. These findings underscore the importance of sediment grain size, water depth and sulfide concentration in amphioxus habitat selection, and highlight the critical role of local anthropogenic pressures and species-specific adaptations in shaping current population dynamics.

In this study, amphioxus identification was conducted using morphological methods. However, molecular approaches such as COI gene DNA barcoding provide distinct advantages in species identification and ecological research. Morphological analysis can offer ecological information including body length, gonadal development, and myotome counts. Yet the highly conserved body form of amphioxus, together with the indistinct features of juveniles or damaged specimens, often results in uncertainty in species determination. In the survey of Dongshan Bay, Fujian, Weng et al. combined morphological measurements with COI barcoding to confirm the presence of *B. japonicum* and revealed a sharp reduction in habitat area and a dramatic decline in population size, providing critical evidence for the establishment of protected areas [18]. Similarly, Guarneri et al. applied DNA barcoding in the North Adriatic Sea to accurately identify populations of *B. lanceolatum* and linked their occurrence with substrate type, density distribution and spawning seasonality, thereby uncovering ecological differences among geographic populations [32]. DNA barcoding not only avoids misidentification arising from morphological similarity but also improves accuracy in cases involving juveniles, cryptic forms, or closely related species. In addition, it provides reliable data for habitat suitability modeling, population genetic structure assessment and conservation planning. Therefore, the integration of DNA barcoding with morphological methods enables complementary and reinforced species identification, offering a more robust scientific foundation for amphioxus resource conservation and ecological restoration.

Furthermore, we focused on the summer season because *B. japonicum* typically enters its peak reproductive period when water temperatures exceed 23 °C, making this a critical life history stage. While our August sampling provides valuable insights into population dynamics during this ecologically significant period, we acknowledge that the single-season approach may introduce seasonal bias and limit the capture of intra-annual variations in habitat use. Future studies incorporating seasonal monitoring will help address this limitation.

While our HSI model effectively captured habitat suitability patterns during the study period, it inherently assumes stationarity in amphioxus’ habitat preferences. That is, the species’ environmental tolerances (e.g., optimal sulfide levels) remain constant over time. This assumption may not hold under scenarios such as Long-term climate change altering thermal tolerances, adaptive shifts in pollution resistance due to chronic exposure, as well as ontogenetic changes in microhabitat use between life stages. Thus, future studies could address this by incorporating time-varying weights through dynamic modeling, validating preference parameters across multi-year datasets, as well as testing for threshold effects in habitat selection.

In addition, the Analytic Hierarchy Process (AHP) is a systematic analysis method that decomposes complex multi-objective decision-making problems into a hierarchical structure consisting of a target layer, criterion layer and indicator layer, and determines the weights of elements through pairwise comparisons. It is widely applied in various fields: in environmental science, habitat suitability evaluation and ecological risk assessment (e.g., determining the weights of key factors affecting amphioxus habitats). Its advantages lie in clear logic, as it can decompose complex problems layer by layer, integrate qualitative and quantitative analysis, convert expert experience into quantitative weights, and has broad applicability to handle unstructured decision-making with multiple criteria. However, it also has obvious shortcomings: it is relatively subjective, as the weights depend on the judgment matrix and are prone to being influenced by experts’ preferences. In this study, we analyzed the relationship between the habitat suitability index (HSI) and amphioxus abundance. Field validation showed that the HSI was in good agreement with the actual amphioxus density measured at 24 stations. Data simulation and field experimental data have effectively enhanced the methodological rigor and the credibility of the results. In future studies, multi-method combination or cross-validation (e.g., combining the Analytic Hierarchy Process (AHP) with the entropy weight method, and conducting comparative validation between the Maximum Entropy Model (MaxEnt) and Generalized Linear Model (GLM)) can be adopted to overcome the limitations of a single method and improve the scientific rigor and reliability of evaluation results.

## 5. Conclusions

In conclusion, this study assessed the habitat suitability of amphioxus in the Changli Marine Reserve and adjacent coastal waters using an HSI model based on AHP. The results identified sediment grain size (Grain 1~0.25 mm), water depth and sediment sulfide content as the primary factors influencing amphioxus distribution. Amphioxus populations fluctuated from 2008 to 2023, with a notable recovery in recent years, particularly within the reserve area, where habitat conditions were generally more favorable. These findings highlight the importance of long-term monitoring and habitat management in mitigating both environmental and anthropogenic pressures.

Specifically, we recommend the following measures to enhance the conservation and management of amphioxus habitats: (1) Monitoring Sediment Quality: Regular monitoring of sediment quality, including grain size and sulfide content, should be implemented to ensure that habitat conditions remain suitable for amphioxus populations. (2) Regulating Aquaculture: Aquaculture activities near key amphioxus habitats should be carefully regulated to minimize disturbances to sediment and water conditions. This includes setting guidelines for the location, scale, and practices of aquaculture operations. (3) Incorporating Amphioxus into Biodiversity Assessments: Amphioxus should be included in national and regional biodiversity assessment frameworks. This would ensure that their conservation needs are considered within broader environmental policies and management plans. These recommendations are aimed at providing actionable guidance for policymakers and conservation managers, thereby increasing the practical value of our research.

In addition, this study was limited to population abundance and environmental factors without integrating genetic or population structure data. Future research incorporating molecular approaches would provide a more comprehensive basis for conservation strategies of amphioxus in northern China.

## Figures and Tables

**Table 1:** The environmental factors significantly correlated with the abundance of amphioxus (*p* < 0.01).

Factor	Correlation Coefficient	*p*	Factor	Correlation Coefficient	*p*
Depth	0.155	0.003	S-TOC	−0.183	0.001
Grain 1~0.5 mm	0.337	0.000	S-sulphide	−0.255	0.000
Grain 0.5~0.25 mm	0.443	0.000	S-oil	−0.276	0.000
Grain 0.125~0.063 mm	−0.442	0.000	COD	−0.200	0.000
Grain < 0.001 mm	−0.216	0.006	NO_2_	−0.208	0.000
Sand	0.215	0.000	NO_3_	−0.150	0.004
Silty sand	−0.216	0.000	DIN	−0.160	0.002
Clay	−0.211	0.000	Chl-a	−0.148	0.007
Grain 1~0.25 mm	0.260	0.000	Transparency	0.235	0.005

**Table 2:** Indicator factor judgment matrix.

Factors	S-Sulphide	Water Depth	Grain 1~0.25 mm
S-sulphide	1	0.6	0.2
Water depth	1.67	1	0.33
Grain 1~0.25 mm	5	3	1

**Table 3:** Habitat suitability index (HSI) of amphioxus at different stations.

Station	HSI	Station	HSI
S01	0.42	S13	0.35
S02	0.31	S14	0.47
S03	0.34	S15	0.40
S04	0.06	S16	0.35
S05	0.30	S17	0.48
S06	0.35	S18	0.51
S07	0.44	S19	0.51
S08	0.30	S20	0.30
S09	0.45	S21	0.36
S10	0.52	S22	0.47
S11	0.52	S23	0.35
S12	0.36	S24	0.38

## Data Availability

If someone wants to request the data from this study, please contact Yongfeng Zhang.

## Supplementary Materials

The following supporting information can be downloaded at: https://www.mdpi.com/article/10.3390/ani15213203/s1, Figure S1: Amphioxus fixed with ethanol; Table S1: Sampling and analytical methods for 50 environmental parameters in seawater and sediments.
