# Supplementary material for: Assessment of Habitat Suitability for Amphioxus in the Changli Marine Reserve and Adjacent Coastal Waters, Hebei Province
Source: Animals (Basel). 2025 Nov 3;15(21):3203. doi: 10.3390/ani15213203 (PMC12609651; doi:10.3390/ani15213203)
Supplement: Supplementary file 1 [file animals-15-03203-s001.zip › animals-3862330-supplementary.pdf]

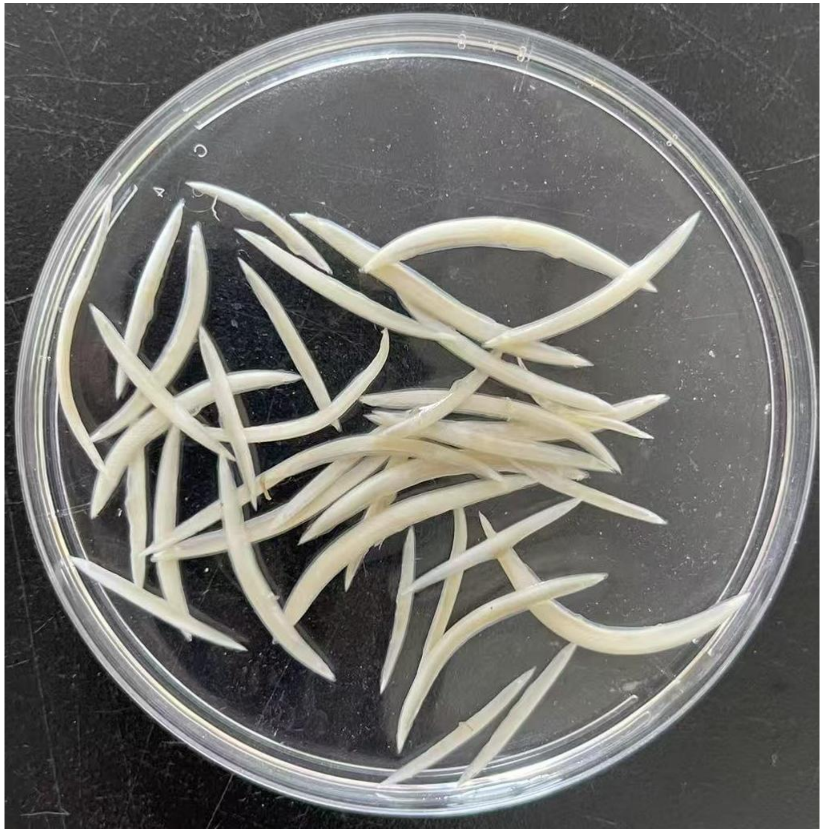

Figure S1 Amphioxus fixed with ethanol.

Table S1 Sampling and analytical methods for 50 environmental parameters in seawater and sediments.

Sample type	Environmental parameter	Sampling method	Analysis method
Water quality parameters	Temperature	Water samples were collected using a water sampler.	Surface water temperature was measured in situ with a thermometer.
	Depth	Water samples were collected using a water sampler.	Depth was determined by a sounding rope.
	Salinity (S)	The sampler was used to rinse sample bottles, fill them, and seal them tightly.	Salinity was measured in the laboratory with a salinometer, calibrated using standard reference solutions, and practical salinity was calculated according to the International Oceanographic Tables.
	Suspended solids (SS)	Water samples were collected with a water sampler, immediately transferred into sample bottles, and shaken continuously to avoid sedimentation, then sealed tightly.	A known volume of water sample was filtered through a 0.45 μm membrane, and the concentration of suspended matter was calculated based on the weight difference of the filter before and after filtration.
	pH	Water samples were collected with a water sampler, sample bottles were rinsed and filled, and then one drop of mercuric chloride solution was added for fixation, mixed well, and sealed.	pH was measured in the laboratory using a pH meter, calibrated with standard buffer solutions close to the expected sample pH, and the field pH value was obtained after temperature and pressure corrections.

	Dissolved oxygen (DO)	Water samples were collected with a water sampler, sample bottles were rinsed with a small amount of water, and water was slowly and evenly introduced into the bottles from the outlet tube placed at the bottom, then sealed after being filled. Immediately, manganese chloride solution and alkaline potassium iodide solution were added in sequence, the bottle was sealed, and inverted repeatedly to ensure thorough mixing of the sample and the fixatives.	The supernatant from the water sample bottle was poured into a conical flask. Then 1 mL of sulfuric acid solution was immediately added to the water sample bottle, shaken until the precipitate was completely dissolved, and transferred completely into the conical flask. The solution was stirred on a magnetic stirrer and titrated with standardized sodium thiosulfate solution until the starch indicator changed color. The DO concentration was calculated based on the volume of titrant consumed.
	Chemical oxygen demand (COD)	The sampler was used to rinse sample bottles, fill them, and seal them tightly.	A known volume of water sample was transferred into a conical flask, and 1 mL sodium hydroxide solution and 10.00 mL potassium permanganate solution were added. The mixture was boiled for 10 minutes, cooled rapidly to room temperature, then 5 mL sulfuric acid solution and 0.5 g potassium iodide were added and mixed. The solution was kept in the dark for 5 minutes, and titrated with standardized sodium thiosulfate solution under continuous shaking, with starch indicator for color development. The COD value was calculated accordingly.

	Dissolved inorganic phosphorus (DIP)	Water samples were collected with a water sampler, bottles were rinsed and filled, and samples were filtered through a 0.45 μm membrane.	A series of suitable phosphate standards was prepared, and 50 mL of water sample was taken for synchronous determination. Then 1 mL of a mixed solution of sulfuric acid–ammonium molybdate–potassium antimony tartrate and 1.0 mL ascorbic acid solution were added, mixed well, and allowed to develop color for 5 minutes. The solution was transferred into a 5 cm cuvette, and absorbance was measured at 882 nm. The phosphate concentration was calculated according to the calibration curve.
	Nitrite (NO2-)	Water samples were collected with a water sampler, bottles were rinsed and filled, and samples were filtered through a 0.45 μm membrane.	A series of suitable nitrite standards was prepared, and 50 mL of water sample was taken for synchronous determination. Then 1.0 mL sulfanilamide solution was added, mixed, and allowed to stand for 5 minutes. Next, 1.0 mL N-(1-naphthyl)-ethylenediamine hydrochloride solution was added, mixed, and allowed to stand for 15 minutes. Absorbance was measured at 543 nm, and nitrite concentration was calculated according to the calibration curve.

	Nitrate (NO_3^-)	Water samples were collected with a water sampler, bottles were rinsed and filled, and samples were filtered through a 0.45 μm membrane.	A series of suitable nitrate standards was prepared, and 50 mL of water sample was taken for synchronous determination. Zinc granules and cadmium chloride solution were added, and the mixture was shaken on a shaker for 10 minutes. The zinc granules were then removed immediately. Next, 0.5 mL sulfanilamide solution was added, mixed, and allowed to stand for 5 minutes, followed by the addition of 0.5 mL N-(1-naphthyl)-ethylenediamine hydrochloride solution, mixed, and allowed to stand for 15 minutes. Absorbance was measured at 543 nm, and nitrate concentration was calculated according to the calibration curve.
	Ammonia nitrogen (NH_4^+)	Water samples were collected with a water sampler, bottles were rinsed and filled, and samples were filtered through a 0.45 μm membrane.	A series of suitable ammonia nitrogen standards was prepared, and 50 mL of water sample was taken for synchronous determination. Then 5 mL sodium hypobromite solution was added, mixed, and allowed to stand for 30 minutes. Next, 5 mL sulfanilamide solution was added, mixed, and allowed to stand for 5 minutes. Finally, 1 mL N-(1-naphthyl)-ethylenediamine hydrochloride solution was added, mixed, and allowed to stand for 15 minutes. Absorbance was measured at 543 nm, and the ammonia nitrogen concentration was calculated according to the calibration curve.

	Dissolved inorganic nitrogen (DIN)	Water samples were collected with a water sampler, bottles were rinsed and filled, and samples were filtered through a 0.45 μm membrane.	Total inorganic nitrogen was obtained by summing nitrate nitrogen, nitrite nitrogen, and ammonia nitrogen.
	Chlorophyll-a (Chl-a)	A volume of 2–5 L of seawater was collected with a water sampler, 2 mL of magnesium carbonate suspension was added and mixed, then the sample was filtered through a 0.45 μm membrane filter, which was stored at low temperature in the dark.	The filter was placed into a centrifuge tube, and 10 mL acetone was added. After thorough shaking, the samples were stored at low temperature in the dark for 14–24 hours, then centrifuged. The supernatant was transferred into a cuvette, and absorbance was measured at 750 nm, 664 nm, 647 nm, and 630 nm. Chlorophyll a concentration was calculated accordingly.
	Transparency	Water samples were collected using a water sampler.	Water transparency was measured in situ using a Secchi disk.
	Oil	A ball-valve water sampler was used to directly fill the sample bottle underwater, which was then brought to the surface immediately and acidified with 5 mL sulfuric acid.	The samples were extracted twice with 5 mL n-hexane solution, the extracts were combined, and absorbance was measured in a 1 cm quartz cuvette at 225 nm. A suitable calibration curve was prepared, and oil concentration was calculated according to the calibration curve.

	Silicate (Si)	Water samples were collected with a water sampler, bottles were rinsed and filled, and samples were filtered through a 0.45 μm membrane.	A series of suitable silicate standards was prepared, and 50 mL of water sample was taken for synchronous determination. Then 3 mL of a mixed solution of sulfuric acid–ammonium molybdate was added, mixed, and allowed to stand for 5 minutes. Next, 2 mL oxalic acid solution was added, mixed, and color was developed until complete and stable. Absorbance was measured at 380 nm, and silicate concentration was calculated according to the calibration curve.
	Copper (Cu), lead (Pb) and cadmium (Cd)	Water samples were collected with a water sampler, bottles were rinsed, filled, filtered, and acidified with nitric acid to pH less than 2.	A series of suitable standards was prepared, and a known volume of water sample was taken. One drop of bromocresol green indicator was added, and the solution was adjusted to light blue with ammonia and hydrochloric acid solutions. Then 1.0 mL ammonium acetate solution and 3.0 mL APDC–DDTC mixed solution were added. An appropriate amount of cyclohexane–MIBK mixture was added, shaken for 2 minutes, and allowed to separate. The organic phase was transferred into a graphite tube, and absorbance was measured by flameless atomic absorption spectrophotometry. Concentrations in water samples were calculated according to the calibration curve.

	Zinc (Zn)	Water samples were collected with a water sampler, bottles were rinsed, filled, filtered, and acidified with nitric acid to pH less than 2.	Prepare a suitable series of standard curves, take a certain volume of water sample, add one drop of dimethyl yellow indicator solution and mix well. Adjust the solution with ammonia until it turns orange-yellow. Add 2 mL of APDC-DDTC-ammonium acetate complexing agent mixture and mix well. Add 3.0 mL of MIBK, shake vigorously for 2 minutes, and allow to stand for phase separation. The organic phase was measured for absorbance by flame atomic absorption spectrophotometry. The concentration in the water sample was calculated according to the standard curve.
	Chromium (Cr)	Water samples were collected with a water sampler, bottles were rinsed, filled, filtered, and acidified with sulfuric acid to pH less than 2.	Prepare a suitable series of standard curves, take a certain volume of water sample, add one drop of dimethyl yellow ethanol solution, and adjust the pH with dilute ammonia or dilute hydrochloric acid until the solution turns light orange. Add one drop of potassium permanganate solution and heat in a water bath for 10 minutes.<br>Add 1 mL of potassium hydrogen phthalate buffer solution and 1 mL of DDTC solution, and mix well. Add 1.50 mL of MIBK, extract for 2 minutes, and allow to stand for phase separation. Transfer the organic phase into a graphite tube, and measure the absorbance by flameless atomic absorption

			spectrophotometry. The concentration in the water sample was calculated according to the standard curve.
	Mercury (Hg)	Water samples were collected with a water sampler, bottles were rinsed, filled, filtered, and acidified with sulfuric acid to pH less than 2.	Prepare a suitable series of standard curves, take a certain volume of water sample into a conical flask, and add 2.0 mL sulfuric acid and 5.0 mL potassium persulfate solution. Heat to boiling for 1 minute, then cool to room temperature. Add 2 mL hydroxylamine hydrochloride solution and mix well. Inject the solution into the hydride generator of a fluorescence spectrophotometer to measure fluorescence intensity. The concentration in the water sample was calculated according to the standard curve.

	Arsenic (As)	Water samples were collected with a water sampler, bottles were rinsed, filled, filtered, and acidified with sulfuric acid to pH less than 2.	Prepare a suitable series of standard curves, take a certain volume of water sample into a colorimetric tube, add 10.0 mL concentrated hydrochloric acid and 2.0 mL thiourea–ascorbic acid reducing agent, and shake well. Leave the mixture for 20 minutes. Take 2 mL of the digested sample solution and measure its fluorescence intensity. The concentration in the water sample was calculated according to the standard curve.
Sediment parameters	Total nitrogen (S-TN)	Surface sediment samples were collected using a grab sampler, placed in polyethylene bags, and stored at low temperature.	The samples were freeze-dried, placed into a Kjeldahl flask, 3.1 g of mixed catalyst was added, followed by 12 mL concentrated sulfuric acid, and digested by heating. After cooling, the samples were subjected to semi-micro distillation. The distillate was titrated with standard hydrochloric acid solution, and the total nitrogen content in the samples was calculated.
	Total phosphorus (S-TP)	Surface sediment samples were collected using a grab sampler, placed in polyethylene bags, and stored at low temperature.	The samples were freeze-dried, oxidized with potassium persulfate under acidic conditions at 120–125°C, and the supernatant was determined and calculated according to the method for measuring reactive phosphate.

	Silicon (S-Si)	Surface sediment samples were collected using a grab sampler, placed in polyethylene bags, and stored at low temperature.	The samples were freeze-dried, and biogenic silica in the sediment was extracted by a two-step method with 0.1 mol/L HCl and 1% Na2CO3 solution. After centrifugation and filtration, the supernatant was analyzed by the silicomolybdate blue spectrophotometric method to determine the concentration of dissolved silica.
	Total organic carbon (S-TOC)	Surface sediment samples were collected using a grab sampler, placed in wide-mouth glass bottles, and stored at low temperature.	Standard curves for total carbon and total inorganic carbon were prepared. For sample measurement, freeze-dried samples were analyzed with a non-dispersive infrared absorption detector to determine total carbon, followed by total inorganic carbon. The total organic carbon content was calculated by subtraction.
	Sulfide (S-sulphide)	Surface sediment samples were collected using a grab sampler, placed in brown wide-mouth glass bottles, flushed with nitrogen gas, sealed, and stored at low temperature.	A weighed amount of wet sediment sample was taken, 3 mL zinc acetate solution and an appropriate volume of water were added, and the mixture was stirred with a glass rod into a slurry. The slurry was transferred into a nitrogen determination apparatus. In a hydrochloric acid medium, sulfides in the sample generated hydrogen sulfide, which was distilled with steam and absorbed by zinc acetate solution to form zinc sulfide precipitate. This precipitate reacted with hydrochloric acid to release hydrogen sulfide, which was oxidized by iodine. The excess iodine was titrated with standardized

			sodium thiosulfate solution. The sulfide content in the sample was calculated.
	Petroleum (S-oil)	Surface sediment samples were collected using a grab sampler, placed in brown wide-mouth glass bottles, and stored at low temperature.	Freeze-dried sediment samples were extracted with n-hexane.<br>The oil content in the extract was determined by ultraviolet spectrophotometry, and the oil content in the sediment was calculated accordingly.
	Mercury (S-Hg)	Surface sediment samples were collected using a grab sampler, placed in wide-mouth glass bottles, and stored at low temperature.	A wet sediment sample was weighed and digested in a nitric acid–hydrochloric acid system in a boiling water bath. Mercury was completely converted to the ionic state in the solution. Under the action of potassium borohydride as the reducing agent, ionic mercury in the solution was reduced to mercury vapor. Argon was used as the carrier gas to transport the mercury vapor into the atomizer of an atomic fluorescence spectrophotometer. A special mercury hollow cathode lamp was used as the excitation source, and the fluorescence intensity of mercury

			atoms was measured. Moisture content was also determined, and the mercury content in the sample was calculated accordingly.
	Arsenic (S-As)	Surface sediment samples were collected using a grab sampler, placed in polyethylene bags, and stored at low temperature.	A low-temperature dried sediment sample was digested in aqua regia solution by heating. After appropriate dilution, hydrochloric acid solution and mixed reducing agents were added. A special arsenic hollow cathode lamp was used as the excitation source, and the fluorescence intensity of arsenic atoms was measured by atomic fluorescence spectrometry.
	Copper (S-Cu), lead (S-Pb) and cadmium (S-Cd)	Surface sediment samples were collected using a grab sampler, placed in polyethylene bags, and stored at low temperature.	A low-temperature dried sediment sample was digested with nitric acid–perchloric acid. In a dilute nitric acid medium, the metals were measured by flameless atomic absorption at 324.7 nm, lead at 283.3 nm, and cadmium at 228.8 nm.
	Zinc (S-Zn)	Surface sediment samples were collected using a grab sampler, placed in polyethylene bags, and stored at low temperature.	A low-temperature dried sediment sample was digested with nitric acid–perchloric acid. The supernatant was analyzed by flame atomic absorption spectrophotometry at 213.8 nm.

	Chromium (S-Cr)	Surface sediment samples were collected using a grab sampler, placed in polyethylene bags, and stored at low temperature.	A low-temperature dried sediment sample was digested with nitric acid and perchloric acid, and chromium was converted to the ionic state. Magnesium nitrate was used as a matrix modifier, and flameless atomic absorption was performed at 357.9 nm.
	Grain size content, grain group content and grain group coefficients (Mdϕ, QDϕ, and SKϕ)	Surface sediment samples were collected using a grab sampler, placed in wide-mouth glass bottles, and stored at low temperature.	A laser particle size analyzer was used for grain size analysis. Grain size parameters were obtained from cumulative probability curves of the particle size distribution. Sediment classification and nomenclature were carried out according to Shepard's triangular diagram of sediment grain size.
